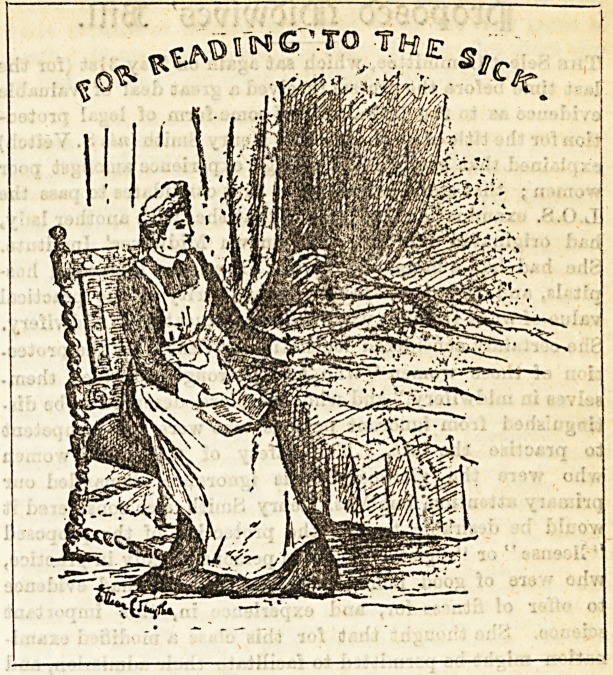# The Hospital Nursing Supplement

**Published:** 1892-06-11

**Authors:** 


"The Hospital" Nursing Mirror.

Being the Extra Nursing Supplement of "The Hospital" Newspaper.

Contributions for this Supplement should be addressed to the Editor, The Hospital, 140, Strand, London, W.C., and should have the word "Nursing" plainly written in left-hand top corner of the envelope.

### En Passant.

QUEEN CHARLOTTE'S LYING-IN HOSPITAL.—The nurses' home of this hospital is now at 232 and 234, Marylebone Road. The fitting up of the home has been a considerable expense, but the success of the new arrangements and the need of it will, we hope, bring more annual subscribers, who will help pay for it.

REDRUTH.—Miss Edith Fry, who was appointed Matron at West Cornwall Miners' Hospital some time since, has also been made Matron at the Women's Hospital, Redruth; the two hospitals though separate in finance and management are connected with each other by a long corridor. It seems a pity they cannot be connected in finance and management as well.

WORCESTER GENERAL INFIRMARY.—Twenty-two probationers were received during the past year at this institution, and we are glad to see that increased and improved accommodation for the nurses and probationers will be provided during the coming year. The authorities have also resolved that the infirmary shall be affiliated with the Royal National Pension Fund for Nurses.

NURSES' WAGES.—The Royal Hants nurses at Winchester, are some of those to whom their hospital "acts justly" to quote from an article in the "Mirror" on the above subject. Many of the nurses at this hospital earn thirty-two pounds a year, they are provided with a full uniform, they get three weeks holiday every year, and they also helped to pay their premiums for pensions in the Royal National Pension Fund; there is not much "sweating" in this case.

ADVANCE GUARDIANS!—It is with great pleasure we learn that the Paddington Guardians have lately given their infirmary nurses a skeleton and Marshall's Diagrams, and they have also completed the medical library for the instruction of the nurses. Dr. Thomas Savill has kindly offered to give courses of lectures, and the Guardians have further ordered that such lectures be made part of the duty of any future Medical Superintendent. A definite course of instruction will help the nurses a great deal, and we congratulate Paddington on such a true mark of progress and enlightenment.

TEA AND TALK.—The new Hospital for Women in Euston Road had a gala day on June 1st, when the whole building was opened to the inspection of a large gathering of visitors, who were entertained with tea and coffee, and received a friendly welcome in the students' library before making the round of the pretty wards. Besides the tastefully-coloured walls of the corridors, the shaded tiles which border the staircase arrest attention; these last were designed by Mrs. Garrett Anderson's sister. In fact this little hospital, where women tend women, is a remarkably dainty and attractive spot, and we cordially congratulate the medical ladies on their successful departure in art as well as in science. The bath-rooms and other sanitary arrangements (shaft-fashion) appear admirable, and the kitchen at the top of the house is large and convenient, whilst the nurses' sitting-room is bright and pleasant, and their little separate bedrooms leave nothing to be desired. There appear to be speaking tubes without number, and handy little "lifts," whilst cupboards, outside the wards, furnish a convenient receptacle for the patients' own clothes which hang therein in orderly array.

MIDDLESEX HOSPITAL.—The Sisters' salaries at this hospital have had a considerable rise, and two of the Sisters have been awarded an annual gratuity of £5 in recognition of the capability shown by them in the training of nurses. The trained nurses institute in connection with this hospital is in a most flourishing condition.

WRENTHAM COTTAGE TRAINING HOME.—Just ten years ago this little undertaking started on its career by opening a small home certified under the Pauper Education Act, by which Guardians are empowered to send girls to be trained for service with a weekly payment not to exceed the cost of workhouse maintenance. First nine girls were taken, and now fourteen girls are received by permission. The object is to give workhouse girls a few months' training, and to provide a home for them between their places so as to avoid their returning to the workhouse. The girls do laundry and needlework, and are taught house work generally. It is really a good work and has, in so far as its intentions have been carried out, fully justified its existence. It rubs off the rough edges of workhouse life, and it has our entire sympathy. Perhaps when workhouses are what we hope they will become, what, in fact, they were originally intended to be, these supplementary homes will be found unnecessary; meanwhile they are doing a good and needed work.

WORK AND HEALTH.—At the Select Committee the other day, Mrs. Scharlieb was asked whether she considered work, such as a medical education entailed, was at all harmful to the health of young women, and she emphatically said that she did not consider it so, and quoted a well-known lady-doctor's vigour and strength. She also asserted that "hysteria and nonsense" were much less prevalent since work, systematic work and study, had become factors in girls' lives. Mrs. Scharlieb's own demeanour was a satisfactory commentary on the statement, for she gave an impression of high training, allied to perfect nerves. She is evidently a good friend to good nurses, and in speaking of them she said that she invariably found those who had fancied beforehand that they should not like working under a woman-doctor were always anxious to take other cases for her after they had had one experience. We were especially glad to hear her testify to the pleasantness and cordiality she had always encountered in her dealings with both nurses and midwives.

A WARNING NOTE.—"Signing an agreement" is a very simple affair, only too easily done and often soon regretted. We wish to warn nurses against blindly committing themselves by putting their names to documents which in many cases they never read through beforehand, and they seldom even ask for a copy of the said document. A case has come to our notice just lately of some nurses who signed to serve an "institution," and in event of their leaving they were prohibited from nursing within thirty miles of the institution. The affair was a "take-in" from beginning to end—indifferent food, indifferent beds, and everything else, and finally the Superintendent announced that she only meant keeping a few nurses, as she did not find a large number "paid," so she turned many nurses adrift, politely intimating that they could go elsewhere. Now is the difficulty of the agreement. The local doctors offer cases, and the nurses hesitate on account of the wretched signature which is held over their heads. We hope to see these particular nurses win their case, as they were "dismissed," and did not leave of their own accord; but it behoves nurses to enquire carefully ere they join a mere money-making concern, and to remember that there are adventuresses of many sorts. All of them are plausible, some are fashionable-looking, others demure, with a saintly air, but they go about and catch the unwary, and generally manage to evade the arm of the law.

### Ventilation, Disinfection, and Diet.

By P. Caldwell Smith, M.D.

### IX.—DISINFECTION—(*continued*).

Creolin or Jeyes Fluid—Condy's Fluid—Sulphurous Acid—Bromine—Chlorine—Chloride of Lime or Bleaching Powder—Osmic Acid—Corrosive Sublimate, the best of all disinfectants—Great disadvantage of its poisonous nature—Antiseptics—Deodorants—Practical disinfection of a room, &c.

Another preparation, derived from Coal tar, which is now being largely used, is creolin, or more popularly Jeyes Fluid, prepared by the Jeyes Sanitary Compounds Company (Limited). It has a tarry smell, and when added to water forms an emulsion like milk. Von Esmarch made a large number of experiments with this substance and found that in most instances it was more efficacious than carbolic acid, more especially with the germs of Asiatic cholera, typhoid fever, and pus. It should be used in 2 or 3 per cent. solution, or very roughly, a tablespoonful to 20 ounces of water. Creolin powder was also found to be fairly effective, as after half-an-hour a 50 per cent. solution of the powder killed all the germs in a putrifying fluid. The soap made with creolin was also tested by him, as it has been recommended for the disinfection of the hands and household objects, and he found that this soap was quite sufficient to destroy putrifactive germs.

Condy's Fluid is a disinfectant which has been used for a long time, but it was found that it required a 5 per cent. solution for 24 hours to destroy the spores of micro-organisms; if it is required to disinfect a solution containing either germs of putrefaction or disease, then this has to be added until it is present to the extent of 5 per cent. of the whole liquid. As Koch says, a 5 per cent. solution of permanganate of potash, which is the active agent in Condy's Fluid, is quite inadmissible for disinfection in bulk.

It has been always the custom for many years to fumigate rooms with sulphurous acid, produced by burning flowers of sulphur. Now this, as ordinarily done, is absolutely useless as far as the killing of the germs of disease is concerned. Koch found that after twenty-four hours exposure to sulphurous acid all the germs which had been placed in the room were quite as active as before, and in his experiments he burnt a very much larger amount of sulphur than is ordinarily done, covering up all the openings into the room very carefully. The sulphurous acid has no penetrating power, so that it will not disinfect any thick material, clothing, or beds.

It has been found that besides permanganate of potash, which we have mentioned already, only perchloride of mercury, or corrosive sublimate, osmic acid, chlorine, bromine, and iodine are rapid as well as sure disinfectants.

Experiments made with bromine in 2 per cent. solution with water, showed that spores were absolutely destroyed within twenty-four hours; with chlorine water, forty-eight hours; while with iodine in alcohol five days were necessary.

Chlorine is a yellowish-green gas, and may be prepared by pouring some HCl, or spirit of salt, on chloride of lime or bleaching powder. One and a half pounds of the latter, and about two pounds of the former should be used for every 1,000 cubic feet. Some experiments made with this gas in swine plague proved that if a stable were fumigated well with chlorine, healthy animals were not infected from diseased ones, and that one good fumigation of a stye in which animals had died of this disease, prevented future occupants from being infected. There can be no doubt that chlorine vapour is more efficacious than fumes of sulphur, but it requires to be carefully handled and not breathed, as it is very irritating and poisonous. This cannot, however, be used for bedding, as it does not penetrate easily.

Bromine vapour has also been used, but it is very destructive to cotton and woollen materials, and it is much more expensive than chlorine.

Whenever a room is to be fumigated, all the window crevices should be pasted up and the fire-place closed. After the vapour begins to come off, the operator should leave the room at once, and paste up the door from the outside. It is well to moisten the air of the room before using chlorine by means of steam.

Osmic acid in 1 per cent. solution is a very efficient disinfectant, but it is a rare substance, and may be placed out of account. There only remains for our consideration the best of all disinfectants, corrosive sublimate. The disadvantage it labours under is that it is extremely poisonous, and its use must be carefully watched.

Koch showed that the spores of that most virulent disease, anthrax, were destroyed in ten minutes by a solution of this substance, in the strength of 1 in 1,000.

Garden earth, which contained the spores of some germs very difficult to destroy, was sprayed with a solution of a strength of 1 in 5,000, and was completely sterilised. It cannot be used for fumigation, as it is not volatile, but a spray can be used in any room for spraying the walls of a strength of 1 in 5,000, which can hardly be said to be poisonous to human beings. To make these quantities more practical, two grains of corrosive sublimate in 20 ounces, or one pint of water, makes a solution of 1 in 5,000, and can safely be used. For washing hands, a strength of 1 in 1,000, or 10 grains in one pint, may be used. This substance can hardly be used for substances containing albuminous matters as, for example, the stools of a typhoid patient, or the sputa of a tuberculous one, and that for a chemical reason: A substance is formed which is not a disinfectant at all, and consequently may lead to a false security.

As previously stated, antiseptics are substances which arrest the growth of germs, and all those true disinfectants already mentioned are also antiseptics. Antiseptics may be used, not so much for the purpose already described by the killing of the germs of infectious diseases, but for the preservation of various articles of food. Cold, when properly applied, is to some extent an antiseptic, as it prevents putrefaction as long as it is applied, as is seen in the importation of frozen mutton, and the transit of fish in ice. Borax, boracic acid, and some other acids, as sulphurous acid, may be used for the same purpose. A large number of so-called essential oils, as oil of mustard, pepperment, cloves, and eucalyptu may also be classed in this category as quinine, alcohol, and salicylic acid.

Deodorants again are used to kill the odours produced during putrefaction. Condy's Fluid, sanitas, creolin, and nitrous acid are good deodorants.

We are now in a position to apply these disinfectants practically, and I shall first describe how to disinfect a room after a disease such as small-pox.

It is not to be expected that there are any carpets or curtains in a room which has been used as a sick room for any infectious disease. All these should be removed at first, and if the patient has been sleeping in that room before coming under treatment, they should at once be disinfected with a steam disinfecting apparatus. If, however, they have not been removed, they should be taken down before applying fumigation, as they are liable to be injured by the action of the agents used. All articles of clothing should also be disinfected in a steam disinfector. As already mentioned, the first thing to do is to paste up all the windows and fireplace. Then small strong bowls with bleaching powder in them are placed at different parts of the room and at differlevels. Strong HCl is then poured into each; the room should be immediately vacated, and the door pasted up so that none of the chlorine fumes can escape. Two pounds of bleaching powder will be found sufficient for an ordinary sized bedroom. The room should not again be entered for at least twelve hours. The best way to enter it is to first, from the outside, open the window or windows as far as possible. To do this, of course, the window sashes should not be fastened. Then the door may be opened, a fire lit, and the bed, bedding, &c., removed and sent for disinfection by steam. All towels and sheets which will stand boiling with a disinfectant should at once be taken to the wash-tub or boiler. I may mention that if there are any metallic surfaces in the room, they should be covered with a coating of vaseline. All the woodwork in the room, chairs (which should not be cushioned), floor, skirting, should be washed with creolin or sublimate solution, the paper stripped off the walls, and the ceiling limewashed. The room may then be ventilated freely for a few days, fresh paper put on the walls, and the furniture may then be put in.

### Asylum Notes.

Fife and Kinross District Asylum, Cupar.—The following members of the staff of this institution have passed the examination of the Medico-Psychological Association, qualifying them for the certificate for proficiency in nursing and attending on insane patients, Miss Burton, Matron; attendants: Allan Grant, James Ness, W. A. Bremner, A. Sontar, George Lumsden, and James Eadie; nurses: Amelia Kennedy, Elsie N. Hadden, Betsy Culbert, Sophia Ballantine, Agnes Taylor, Jessie Bonthrone, Eliza Honeyman, Margaret Kirkcaldy, Lilias Ames.

Derby Borough Asylum.—On May 27th, an interesting function was held at the asylum, when the Mayor (Councillor T. H. Harrison) presented the certificates to the nurses and attendants who had passed the examination of the Medico-Psychological Association. The examination was conducted by Dr. Macleod, of East Riding Asylum, Beverley. The Mayor at the same time presented prizes gained at examinations on lectures on special and general nursing, held during the winter by the medical officers of the asylum. Certificates were gained by Miss R. Sutton, chief nurse; Louisa Asbury, nurse; Elizabeth Macaulay, nurse; Elizabeth Milne Withers, nurse; Eliza Woollatt, nurse; John Guttridge, attendant; and William Guttridge, attendant. Prizes gained by (1) Ida Adam and Helen Macdonald, (3) Eliza Woollatt, (4) Grace Gomm (nurses), and William Guttridge (attendant), and (6), Mary Bostock (nurse).

### Presentations.

Withington Workhouse Hospital.—On Friday last, Nurse Ditchfield was presented with a travelling bag from the nursing staff, and with a handsome satchel and umbrella from the other officers of the institution. The presentation was made by one of the resident medical officers, and was given as an expression of goodwill towards Nurse Ditchfield, who, after 12 years' work as probationer, charge-nurse, and midwife, at the Withington Hospital, is about to leave for America.

Edinburgh City Hospital.—On the 18th ult. Dr. A. Fleming-Wood, Medical Superintendent of this institution, was presented by the nursing staff with a handsome travelling dressing bag. Seven years ago the old Infirmary of Edinburgh was taken over by the City authorities, and converted into a City Hospital for the treatment of infectious diseases. Dr. A. Fleming-Wood, who had been for seven years in charge of the fever wards, was appointed Medical Superintendent. On the completion of his seven years' service the opportunity was taken to show the respect in which he was held by those who came most in contact with him. Councillor Pollard, Convenor of the Health Committee, made the presentation on the nurses' behalf. Dr. Wood, in accepting the gift, said that it was just fourteen years ago since he entered the Fever Hospital, and during that time his life had been a very pleasant one, thanks to the universal friendship and kindness that had reigned in the community.

### THE SHORN LAMB.

There is a true and pathetic story told by a celebrated writer travelling in France in the last century, about a young girl, "poor Maria" he calls her, who, deserted by her lover, and with a mind unhinged by her misfortune, wandered in search of him as far as Rome and back. She had found her way alone across the mountains, travelled all over Lombardy without money, and through the flinty roads of Savoy without shoes. How she had borne it, and how she had been supported she could not tell. To all inquiries her answer was, "I do not know, but 'God tempers the wind to the shorn lamb'"; these words had consoled her in all her wanderings.

How often we poor, disconsolate, sensitive creatures shiver in the strong wind of God's reproof like lambs just released from the hands of the shearer. We have lost our health, our money, our loved ones, and, like this girl, would go anywhere to regain them, and happy is it for us if we can, in our fancied wisdom, find the comfort which her poor crazed brain had grasped, namely, that our heavenly Father knows how much we can bear, and softens the blast of His correction to our needs. Fear not, be strong, Christ is very tender to the weak. The prophecies about Him in the Old Testament, and all the works recorded of Him in the New, point out this gracious quality. In the former He is spoken of as a refuge from the storm, a shadow from the heat, a hiding place from the wind, and the shadow of a great rock in a weary land; while He says of Himself that He came to preach good tidings to the meek, to bind up the broken-hearted, to proclaim liberty to the captives and the opening of the prison to those that are bound. Are there any of us children of sorrow to whom these promises will not apply? Poor, lonely, sick, in bondage to Satan, blind in mind, and withered in heart, we may come to Him for strength and protection, for "a bruised reed shall He not break, and the smoking flax shall He not quench. He will fan into life the faintest gleam of spiritual desire, and the once bruised reed shall resound with the voice of melody."

While Christ trod this earth we know how tender he was to sinners. Towards His disciples, from whom He had to bear so much His voice never rose into harsh upbraidings, and notwithstanding that St. Peter denied Him in His last agony, and the rest forsook Him and fled, yet the message sent by Him through the angel to the women at the Sepulchre was full of love and clemency. "Go tell His disciples and Peter that I am risen." And to those outside His own little band of followers He was no less thoughtful and tender; the penitent woman in Simon's house was pardoned and comforted, as was also she who came to Him in His thirst at the Well of Jacob, while He pitied and relieved the fainting multitudes in the wilderness who had come from afar.

### Proposed Midwives' Bill.

The Select Committee, which sat again on May 31st (for the last time before the recess) received a great deal of valuable evidence as to the desirability of some form of legal protection for the title of midwife. Mrs. Henry Smith (*née* S. Veitch) explained that she had had a large experience amongst poor women; that she was one of the first candidates to pass the L.O.S. examination (1873); and that she, with another lady, had originated the now well-known Midwives' Institute. She had had a large and varied experience in general hospitals, and therefore spoke with authority of the practical value of ward training when allied to education in midwifery. She certainly considered legislation advisable for the protection of those women "who had thoroughly trained themselves in midwifery," and who, therefore, deserved to be distinguished from ignorant persons who were not competent to practise the art. The safety of the poor women who were the victims of this ignorance demanded our primary attention, but Mrs. Henry Smith also considered it would be desirable to give the protection of the proposed "license" or "register" to those persons already in practice, who were of good moral character, and who had evidence to offer of fitness for, and experience in, this important science. She thought that for this class a modified examination might be permitted to facilitate their admission, and that in all cases a guarantee of capacity from the doctors they had worked for, must be demanded. She entirely agreed with Dr. Playfair that "education taught a midwife her ignorance," and hence proper training ensured her seasonable summons of the qualified medical man or women whenever necessity arose. Dr. Edmonds next gave evidence, and said that he considered a "State examination" in the science and practice of midwifery was desirable. He should like the curriculum to extend over twelve to eighteen months, but he did not insist upon this period, although he wished the training to "include some auxiliary subjects," and would like midwifery to be entirely separated from general medical practice; he considered this would be for the benefit of doctors and patients. He a'so propounded various interesting and original theories as to the amount and kind of education desirable, which elicited the opinion from Dr. Farquharson that the person thus treated would be "a half-educated medical woman, not a midwife at all!" and we are inclined to endorse this decision. However, there could be no doubt of Dr. Edmonds' kindly interest, nor of the good opinion he holds of a woman's aptness and ability for this branch of practice, and he spoke regretfully of the "very small and contemptible opposition" offered by some few men to the present scheme." We cannot agree with him as to clergymen being proper supervisors of midwives in count y districts, for doctors must certainly be fitter judges, and they only should be referred to in such matters. Mrs. Scharlieb, M.D., was heard with marked attention, for her evidence was an intellectual treat, as well as particularly valuable in the cause of which few are better qualified to speak than herself. She gave some details respecting the education and subsequent practice of fully-qualified medical women in India, as well as England, and spoke pleasantly of the increasing cordiality with which they were treated by "our medical brethren," from whom she personally had "always received the greatest courtesy." As out-patient physician to the new hospital for women in Euston Road, she saw about a hundred women, twice a week, and often met with cases, the direct results of injury inflicted by ignorant women, she believed unconsciously, but the fact was none the less serious and frequently none but palliative measures were possible. Mrs. Scharlieb spoke of the good work done by thoroughly trained midwives in India, and explained the official registration in force there; all certificates of character and experience, as well as diplomas being entered and proper supervision being thus strictly ensured.

She was certainly in favour of legislation for protection of midwives in England, and thought a scheme feasible for admitting competent women, already in practice, to the same register as persons holding the L.O.S. diploma, provided they passed an examination within a defined period, such as twelve months, to prove their efficiency. Regarding the word "Register," to which exception had been taken, Mrs. Scharlieb saw no objection to the term "Licensed Midwife" if this name would appease the small general practitioner, but whatever the title chosen, it must be a recognised one. On being questioned respecting the requisite education, she said she thought the L.O.S. examination satisfactory, but she distinctly approved of the addition of twelve months' hospital training in general nursing, whenever this was possible. Dr. Champneys agreed with previous speakers that a proper training in midwifery should be followed by an examination, and that the educated midwife should be certified by a proper board or examining body. He thought there should not be too much decentralisation, for if there were an excessive number of places for examination, the standard might become undesirably lowered in small districts; he thought fifteen centres in the larger towns would meet the case, and might be worked under the Medical Council. He considered the knowledge required by the L.O.S. was accurate, and that their examinations were satisfactory, and he certainly approved of general training in nursing as an addition to teaching in midwifery. He considered that experienced nurses appreciated the value of antiseptics and perfect cleanliness, as well as any doctor. Questioned with regard to alleged competition between doctors and midwives, he said "There was none when the practitioner was capable." He thought there were three classes of men: those who objected to losing small fees, those who were unwilling to lose practice and experience, and those who were unfeignedly glad to resign a troublesome and badly-paid branch of practise entailing disturbed nights and loss of much time; he fancied the first and second were eventually merged in the third class. In all cases of difficulty, the properly-trained midwife could be absolutely trusted to summon the doctor at once. He was also of opinion that she could do more for the woman, in ordinary cases, than the over-worked general practitioner, who wanted to be in half-a-dozen places at once. With regard to the fear of competition expressed by certain doctors, Dr. Champneys' opinion was short and to the point, for, said he, "A man can soon find out whether the public wants him, and if it does not, why let him put up his shutters and go," not try to create a demand.

### Notes and Queries.

### Answers.

*Hospital Reader.*—1. Certificate earned in Children's Hospital would be of no value for general nursing purposes. 2. Training in general nursing includes treatment of children. 3. Write to Matrons of the hospitals you name, for forms of application for probationers.

*Cymro Anwyh.*—Only nurses already trained are eligible at military hospitals. Write to Matrons of the Hospital and Dispensary, and the King's Cliff Hospital, both at Scarborough.

### Wants and Workers.

[Under this heading, we propose to try whether we can be useful to our readers in making the wants of some known to others who are willing to do what work they can to aid the great cause of curing and cheering the sick. Wants can only be inserted from those who are connected with some institution or association, or who are willing to have their full name and address printed.]

*Miss Isabelle Linnell* writes to say that a lady has written kindly offering a piano for Foulis Ward, Brompton Hospital.

*Miss S. A. Wood, Children's Convalescent Home, Ramsgate,* wishes to thank the kind friend who has sent a spinal carriage for an invalid child. If anybody has an invalid chair to spare, Miss Wood would be very grateful.

### Everybody's Opinion.

*[Correspondence on all subjects is invited, but we cannot in any way be responsible for the opinions expressed by our correspondents. No communications can be entertained if the name and address of the correspondent is not given, or unless one side of the paper only be written on.]*

### THE NEEDLESS DREAD OF NURSING SMALL-POX CASES.

"J. M." writes: From personal experience I can endorse what was said in last week's number about temporary hospitals being unsatisfactory or inefficient. Panic legislation is always bad legislation, and this is exemplified in more than one town in the North, where inadequate and unseaworthy sheds—no less—have been run up for the accommodation of small-pox patients. Laying the blame of bad legislation then on our local parliaments, we will look at the result of their management. The nursing is relegated to untrained and ignorant agents. I need not amplify, for in doing so I might seem to hold up to ridicule these agents, and to do so were most unfair. Why is this small-pox nursing so relegated? As to its nursing. A severe case of a bad type calls for great refinement and nicety, and, in case of complications, it wants the same amount of general knowledge and skill in applying it as is common in all cases, but no more. The amount of "nursing" which the worst case of small-pox takes is not at any time equal, I consider, to a very bad typhoid case. We hear a great deal about the loathsome character of small-pox. Let us try to imagine any of our general hospitals or our permanent fever hospitals in ignorant, untrained hands, and I think we should not have to search far for "loathsome" examples. As to the chances of contracting the disease, this is a point on which one should be sure before making any assertions; but, without asserting, I should like to say that, on inquiring, I believe it would be found that the chances of taking small-pox, if it is nursed on enlightened lines, are not greater than with any other infection. In fact, I want to make a point that, under good structural conditions and in competent trained hands, a case of small-pox need be as little dreaded as any other kind of fever. So the conclusion we arrive at is that our authorities are to blame for the manner in which many of our temporary small-pox hospitals are conducted, because they neglect to provide against a panic, and instead furnish "ludicrously inadequate" temporary hospitals without the space and convenience which we nurses consider to be as essential to our work as a clear code of morals. The dread of nursing this fever belongs to many years back; so that if trained nurses do not come forward freely to grapple with the epidemic, it must be the fault of our Local Government Boards and their "fitful, panic-stricken action."

### PRIVATE NURSES AND THEIR WAYS.

"H. F. G." writes: In answer to the correspondent who writes, "How is it that we are always hearing complaints of private nurses nowadays," I should like to say that I have just returned from a visit to a relation who is seriously ill, needing nursing of the most skilful order. In the momentary absence of his attendant he took occasion to say, "It would be impossible to have a better nurse than this one." Ten days ago the physician summoned her, and the sick man's wife waited tremblingly for the awful apparition of the trained nurse (probably she had heard of some of the specimens "V. M. H." has spoken about). In the shortest time possible a gentle ring at the bell heralded the arrival of a quiet-looking woman, whose calm face and sweet voice at once inspired confidence. She was soon in possession of the sick room, in her pretty cap and apron, and had made her patient's acquaintance, carried out written orders left by the doctor, encouraged the sad wife and nervous servants, and, quite unconsciously, had convinced the invalid that, if human help could avail, her strong yet tender hands were quite capable of holding him back from the very gates of death. If, alas! there must be merited blame, let there also be just praises of our much-criticised private nurses.

### ST. OLAVE'S INFIRMARY, ROTHERHITHE.

"ONE WHO HAS BEEN THERE" writes: Perhaps a few facts about St. Olave's Infirmary, Rotherhithe, may be interesting to your numerous readers. I saw in THE HOSPITAL a ground plan of the infirmary, which allow me to say, is not quite a correct one; for instance, the bath-rooms are not double ones, but they are very well arranged. The bath-room, being a large one, a portable bath stands there when not in use, and when it is wanted it is wheeled beside a bed, or into the separation wards. Again, the w.c.'s in the nurses' home do not lead directly off the main corridor, but are approached by a second narrow corridor. I think a visit to our infirmary will shew that it is not built on a low standard, but will vie with any hospital or infirmary in London or the provinces for the arrangements of the wards, ventilation, heating appliances, and appointments. I think, too, a visitor would not find the beds arranged in pairs, but each at an equal distance apart. You will also be glad to hear that the whole of the nursing staff is being re-formed and re-organised. As at Marylebone, a flat consisting of two wards and one or two separate wards, 60 to 64 patients in all, is now ruled by a charge nurse or Sister—who is a thoroughly trained certificated nurse—several of our large London hospitals being here represented. The Matron is also a trained nurse, and has spent some time and energy in endeavouring to improve the tone, and also the nursing itself. So far her efforts have been rewarded with some considerable success. The patients have certainly benefited by the change of having trained nursing, and look happy and contented. The nurses, judging by their faces, look happy too, and seem thoroughly interested in their work. The arrangements of the nurses' home are excellent—on one side it overlooks Southwark Park, on the other the infirmary grounds (now being laid out); green trees and flowers are very refreshing to look upon when tired. Each nurse has a separate bedroom, the assistant nurses and probationers sleep in small rooms and dormitories in the old block. The food, though plain, is good. The Chairman of the Committee enquiring the other day if the nurses had any complaints to make, elicited the reply they had none, though they would like more variety. The nurses get half a day's leave once a fortnight, and a whole day once a month, and on this particular day are allowed the luxury of breakfast in bed. St. Olave's, Rotherhithe, has certainly made many steps in the right direction, and many thanks are due to the Medical Superintendent for his lectures in the past, and great credit is due also to the Matron who has worked so hard to improve the condition of this one of our largest London infirmaries.

### The Nurses' Bookshelf.

### NURSING AND HYGIENE.*

THE second edition of this excellent book should be welcomed, not only by nurses and probationers, but by all who are interested in these two closely-allied subjects, which are certainly here dealt with in a very thorough and simple fashion. The illustrations are numerous and good, and are likely to prove very helpful to the student of these attractive pages. The ventilation and position of a sick-room are most carefully explained, and so are the different important points respecting the feeding and moving of patients. The proper method of washing the person is dealt with in a plain yet detailed way which must prove valuable to readers who have not the opportunity of receiving practical teaching in a hospital ward. Contagion and infection receive the attention demanded by the seriousness of their characters, and subject of "pain," in various aspects, is enlarged upon, as well as systematic "observation" of sick people. Not only is the administration of medicines very clearly explained, but different forms of measuring glasses are capitally shown by illustrations. Bandaging, poultices, the clinical thermometer, and inhalations are amongst the subjects treated of in this practical volume, and we certainly feel surprised at the number of useful things which are compressed into such a moderately-sized book. With regard to the matter of the nourishment being offered in proper, not excessive, quantities, Dr. Roberts remarks, "Enough is as good as a feast, in this instance, a great deal better." There is sound advice in the paragraph. Food should never be kept in the sick-room, so if a patient cannot finish what you bring him, take away the remnant at once. The sight of food, except at the proper moment, disgusts the invalid; it is seeing "too much of a good thing." Baths, packs, and steam baths are well explained, and the sketch of the latter is really a first-rate one.

* "Illustrated Lectures on Nursing and Hygiene." By R. Lawton Roberts, M.D., Lond., D.P.H. Camb. Price 2s. 6d. (Publisher, H. K. Lewis.)

### Four Months in a Hospital Ward.

### A PERSONAL EXPERIENCE IN A PROVINCIAL HOSPITAL—IV.

### Our Nursing Staff.

At the head of the nursing department was a charge nurse, an official who had private quarters on the corridor adjacent to the ward, and upon whom the whole nursing responsibility devolved; under her were the night nurse, also an official, with quarters in a separate building within the grounds, and two probationer nurses, who were changed from ward to ward at intervals of about a month. These probationers were practical students of the art of nursing, and were of two kinds, one engaged for two years at the hospital itself, and one engaged for three years by some outside nursing institution, the proprietors of which send their members for the first year to a hospital to be trained, and for the remaining two years send them out to private nursing. We could distinguish these two kinds by a difference in their uniforms.

### Dainty Appearance of our Nurses.

Not a little of the cheerfulness of our surroundings was contributed by the bright dainty appearance of our nurses, with their snowy white square muslin caps, bibbed aprons, and chatelaines. The charge nurses were distinguished by two long streamers from the back of the cap. The probationers come on duty at seven o'clock in the morning, having already breakfasted, and a hard day's work lies before them. Up till nine o'clock they are furbishing, and from then till noon, when they dine, their attendance upon the patients under the guidance of the charge nurse, keeps them closely employed. Every alternate afternoon they are allowed off duty for about three hours, and even this relaxation does not prevent a frequent breakdown, especially during the first months of their probation, showing how severe is the strain of hospital work upon the unseasoned. From four to nine o'clock they are again busily employed, especially during the last hour when settling down the patients for a night, and then, tired out, they quietly slip away to their own well-earned and much needed rest. All the probationers have one room in common, called the day-room, for their use when off duty; it is situate at the top of the building where also is their dormitory. The severity of the work, of course, varies with the number and nature of the cases in the hospital at the time being; at one time in our ward we had as many as five "typhoid fevers," which, being a non-infectious fever, were always treated in the medical ward, and as the high temperature in some of them necessitated an almost hourly application of iced water for fifteen minutes or more, you may form some idea of the labour entailed upon these probationers. In this hospital, I believe, there are eight departments, all conducted in a similar manner to that in which I lay, viz., men's medical, men's surgical, men's accident, women's medical, women's surgical, women's accident, the children's ward, and the fever block, in all about two hundred and thirty beds, the last for infectious diseases only, and completely isolated from the rest of the hospital both structually and in the matter of the nursing staff.

### No Quarter from the Enemy.

Every probationer had to serve a turn of one or two months in this "fever block" at some period of her training, being during that time entirely cut off from all intercourse with other than her fellow nurses in that department. In view of the grave risk to their own lives thus fearlessly entered upon, I do not think it is too much to say that they they are as deserving of praise and honour as is the soldier, who, forgetting all else but his duty, rushes bravely into action for the good of his country; with the difference here, in the probationer's courage, that there is none of the pomp and circumstance of war to spur onwards to deeds of valour; no open enemy in proud defiant array to vanquish and overcome; only a subtle insidious foe floating in the secure ambush of an invisible atmosphere, whose mortal thrust is delivered beyond all power of parrying, even while its fresh victim is tending the wounded who have already fallen at the hand of the same dread enemy. For the soldier there is an international code of warfare, for the nurse with her enemy it is "no quarter." These eight departments, added to the occasional cases requiring one nurse's sole attention night and day, will show you how great a help to economical management is the assistance of these recruits. The resident doctors give lectures frequently on medical subjects to the probationers, who write papers afterwards thereon for the doctor's correction, and as the probationers are constantly moved about from ward to ward, by the time their training is at an end, they have had a pretty good insight into the mysteries of nursing.

### All Women are not Nurses Instinctively.

It is a popular error to suppose that every woman is instinctively a nurse. It is not so; the germ of a special gift may be there, but nursing has to be learnt, as has every other kind of knowledge, and the sympathetic labouring that passes current as such, in private life, only handicaps the patient and retards his recovery. That there are some ladies at home who can nurse, I readily grant, but they are few and far between. Not for a moment would I ridicule the heart broken grief with which a fond wife or mother views and endeavours to relieve the suffering and helplessness of one they hold dearer than life, and happy is the man who, in addition to a trained nurse, can have the comfort and solace of a loving face at his sick bedside. I only desire to point out the immense advantage to a patient, be he rich or poor, in skilled over unskilled nursing, and then leave your mind to grasp what it means to a poor man, almost broken by the storm, suddenly to find himself in this harbour of refuge and repair.

### A Vigorous Wardmaid.

In addition to the nurses there is attached to each ward a ward maid, whose duties are somewhat of the domestic housemaid character, and include such details as making up the fires, blacking grates, and scrubbing the floors, &c., but who has nothing to do with the patients. The work of this post is heavy, especially in the morning, and it would astonish an average "domestic" to see the activity and "go" of our ward maid, slipping about on her knees like a vigorous eel, with a whisp of tow in her hand, and a pan of bees'-wax sliding along before her like a Scotch curling stone on the ice, and then pounding away with a fourteen pound scrub until she could fairly have seen the reflection of her own honest face in the floor she worked upon. We were all very fond of our ward maid, a yellow haired black country girl of sturdy build, who loved flowers, a rose above all, and who wore a "fringe" on Sundays. Not one of the convalescents who had the strength to wield a scrub, but who would cheerfully keep time with this good natured worker from end to end of our long ward, and out on to our share of the corridor, just to help her a bit, you know.

It only remains now, in detailing the plan of management, to mention the Night Superintendent, whose duties appeared to be a general supervision of the night nurses during their hours of duty. Often when I have lain awake through those few dark hours before the dawn, so terribly long to a sick man, have I seen this tall figure, enveloped in a shawl, glide slowly in and out of the ward like a weird ghost, without as much as the sound of a footfall; in the morning she signs the night nurses' report, and then retires with her band of night watchers at nine o'clock to their quarters in the grounds. The night nurses dine directly they go off duty, and then go out until about noon, at which time they return and sleep until about seven o'clock in the evening, when they have breakfast.

(*To be continued.*)

## Figures and Tables

**Figure f1:**